# Radiomics-Based Artificial Intelligence and Machine Learning Approach for the Diagnosis and Prognosis of Idiopathic Pulmonary Fibrosis: A Systematic Review

**DOI:** 10.7759/cureus.87461

**Published:** 2025-07-07

**Authors:** Asma Khalid, Muhammad Muaz Mushtaq, Saba Sattar, Yan Naing Soe, Sulman Ismail, Muhammad Haris, Sami Ullah, Muhammad Wali Hassan, Muhammad Muaz Bhatti, Husnain Ali

**Affiliations:** 1 Internal Medicine, King Edward Medical University, Lahore, PAK; 2 Medicine and Surgery, King Edward Medical University, Lahore, PAK; 3 Diabetes and Endocrinology, University of Plymouth, Plymouth, GBR; 4 Internal Medicine, Akhtar Saeed Medical and Dental College, Lahore, PAK; 5 Medicine, King Edward Medical University, Lahore, PAK

**Keywords:** artificial intelligence, computed tomography, computer-aided diagnosis, deep learning, idiopathic pulmonary fibrosis, interstitial lung disease, machine learning, radiomics

## Abstract

Idiopathic pulmonary fibrosis (IPF) is a devastating interstitial lung disease (ILD) characterized by progressive fibrosis and poor survival outcomes. Accurate diagnosis and prognosis remain challenging due to overlapping features with other ILDs and variability in imaging interpretation. This systematic review evaluates the current evidence on artificial intelligence (AI) and machine learning (ML) applications for the diagnosis and prognosis of IPF using computed tomography (CT) imaging. Following Preferred Reporting Items for Systematic Reviews and Meta-Analyses (PRISMA) guidelines, eight studies published between 2017 and 2024 were included, demonstrating promising results across various methodologies, including deep learning (DL) models, support vector machines (SVMs), and ensemble approaches. AI-derived parameters, particularly measures of fibrotic burden and pulmonary vascular volume, consistently outperformed conventional visual CT scores for prognostication. Strong correlations between AI-quantified CT features and pulmonary function (PF) tests suggest potential surrogate markers for physiological parameters. Novel prognostic biomarkers identified through AI analysis expand understanding beyond traditional parenchymal assessment. Despite these advances, limitations include retrospective designs, sample size constraints, male-predominant cohorts, and limited external validation. Future research should prioritize large, prospective, multi-center studies with diverse populations, standardized protocols, explainable AI (XAI) techniques, and integration into clinical workflows to realize the transformative potential of AI for improving IPF management.

## Introduction and background

Idiopathic pulmonary fibrosis (IPF) is a chronic, progressive, and ultimately fatal interstitial lung disease (ILD) of unknown etiology, primarily affecting older adults [1]. IPF is characterized by the relentless scarring of lung parenchyma and a usual interstitial pneumonia (UIP) pattern, leading to irreversible decline in pulmonary function (PF) and poor survival outcomes, with a median survival of three to five years post-diagnosis [2]. Early and accurate diagnosis is critical for timely therapeutic intervention, especially with the advent of anti-fibrotic agents that may slow disease progression. However, diagnostic uncertainty remains a major challenge due to overlapping features with other ILDs and variability in imaging interpretation, particularly on high-resolution computed tomography (HRCT) scans. HRCT has become a cornerstone in the evaluation of suspected IPF, often obviating the need for invasive lung biopsy when classic UIP patterns are present [3]. Yet, the interpretation of HRCT images is largely subjective, depending heavily on radiologist expertise and experience. Interobserver variability, even among ILD specialists, complicates consistency in diagnosis and staging. Furthermore, the prognostication of IPF - predicting disease progression, exacerbation risk, or survival - remains difficult with conventional imaging and clinical metrics alone. As the field of pulmonary medicine embraces precision diagnostics, there is growing interest in leveraging artificial intelligence (AI) and machine learning (ML) to enhance the accuracy and reproducibility of IPF diagnosis and prognosis using CT imaging.

AI, particularly its subdomain ML, refers to computational systems capable of learning from data to identify patterns, make decisions, and improve performance over time without explicit programming. In medical imaging, ML algorithms - including convolutional neural networks (CNNs), random forests, support vector machines (SVMs), and deep learning (DL) models - have shown promise in analyzing complex radiological features beyond the capabilities of human perception. For IPF, these tools may not only assist in the automated detection of UIP patterns but also quantify subtle imaging biomarkers such as texture, volume loss, and honeycombing distribution that are predictive of disease severity and outcome [4]. Several recent studies have explored the utility of AI-based models in classifying ILD subtypes, differentiating IPF from non-IPF ILDs, and predicting functional decline or mortality [5]. Radiomics, a process of extracting a large number of quantitative features from medical images, combined with AI algorithms, has further enhanced our ability to derive prognostic signatures from HRCT scans [6]. Moreover, advancements in explainable AI (XAI) are addressing the “black-box” nature of DL, allowing clinicians to interpret the rationale behind algorithmic decisions, and thereby improving clinical trust and integration.

Despite these advances, the current landscape of AI/ML applications in IPF imaging is fragmented. Many studies use small, institution-specific datasets with limited external validation, varying algorithms, and inconsistent reporting standards. This heterogeneity poses significant challenges in assessing the clinical readiness and generalizability of these technologies. Additionally, the ethical implications of AI, including algorithmic bias, data privacy, and regulatory oversight, warrant careful consideration before clinical implementation. Given this rapidly evolving and complex field, a comprehensive synthesis of existing evidence is needed to evaluate the diagnostic and prognostic performance of AI and ML tools in IPF using CT imaging. This systematic review aims to critically appraise and summarize the current literature on AI/ML-based approaches for the diagnosis and prognosis of IPF using CT, highlighting key methodologies, performance metrics, limitations, and future directions. By providing an evidence-based overview, this review seeks to inform clinicians, researchers, and stakeholders about the utility, challenges, and translational potential of AI technologies in the management of IPF.

## Review

Materials and methods

This systematic review was conducted in accordance with the Preferred Reporting Items for Systematic Reviews and Meta-Analyses (PRISMA) 2020 guidelines [7].

Study Selection

A comprehensive and systematic literature search was conducted across multiple electronic databases, including PubMed, Scopus, Embase, and Hinari, from January 1, 2015, to March 1, 2025. The search strategy combined MeSH terms and relevant keywords such as “idiopathic pulmonary fibrosis,” “IPF,” “artificial intelligence,” “machine learning,” “deep learning,” “computed tomography,” “CT imaging,” “radiomics,” “diagnosis,” and “prognosis.” The search was limited to studies published in English. Duplicates were removed using reference management software, and titles and abstracts were independently screened by two reviewers to identify potentially eligible studies. Full texts of relevant articles were then retrieved and assessed for final inclusion based on predefined criteria. Discrepancies between reviewers were resolved through discussion or consultation with a third reviewer.

Eligibility Criteria

Studies were included if they met the following criteria: (1) original research articles involving patients diagnosed with IPF; (2) studies utilizing AI, ML, or DL algorithms to analyze CT or HRCT images for the purpose of diagnosis, classification, severity assessment, or prognostication of IPF; and (3) studies reporting performance metrics such as sensitivity, specificity, accuracy, area under the curve (AUC), or other quantitative outcomes relevant to diagnostic or prognostic evaluation. Exclusion criteria were: (1) reviews, editorials, commentaries, conference abstracts, or case reports; (2) studies not using CT imaging as a primary input modality; (3) studies not focused specifically on IPF or those including mixed ILD cohorts without IPF-specific data; and (4) articles without sufficient methodological detail or outcome reporting to permit data extraction.

Data Extraction

A standardized data extraction form was developed using Google Sheets (Google, Inc., Mountain View, CA, USA). Two reviewers independently extracted data from the included studies, capturing information on study characteristics (authors, year, country, and study design), patient population, imaging modality and acquisition details, AI/ML model type, training and validation methodology, outcome measures (diagnostic accuracy and prognostic indicators), and key findings. Any disagreements in data extraction were resolved through consensus.

Data Analysis

Due to expected heterogeneity in AI model types, study designs, and outcome reporting, a qualitative synthesis of the data was conducted. Studies were grouped based on their primary aim - either diagnostic or prognostic application - and further categorized by algorithm type and validation method. Key performance metrics were summarized narratively and in tabular format to compare the effectiveness and clinical applicability of different approaches. Quantitative synthesis (meta-analysis) was not performed due to methodological variability. Trends, strengths, and limitations were discussed qualitatively.

Results

Study Selection Process

**Figure 1:**
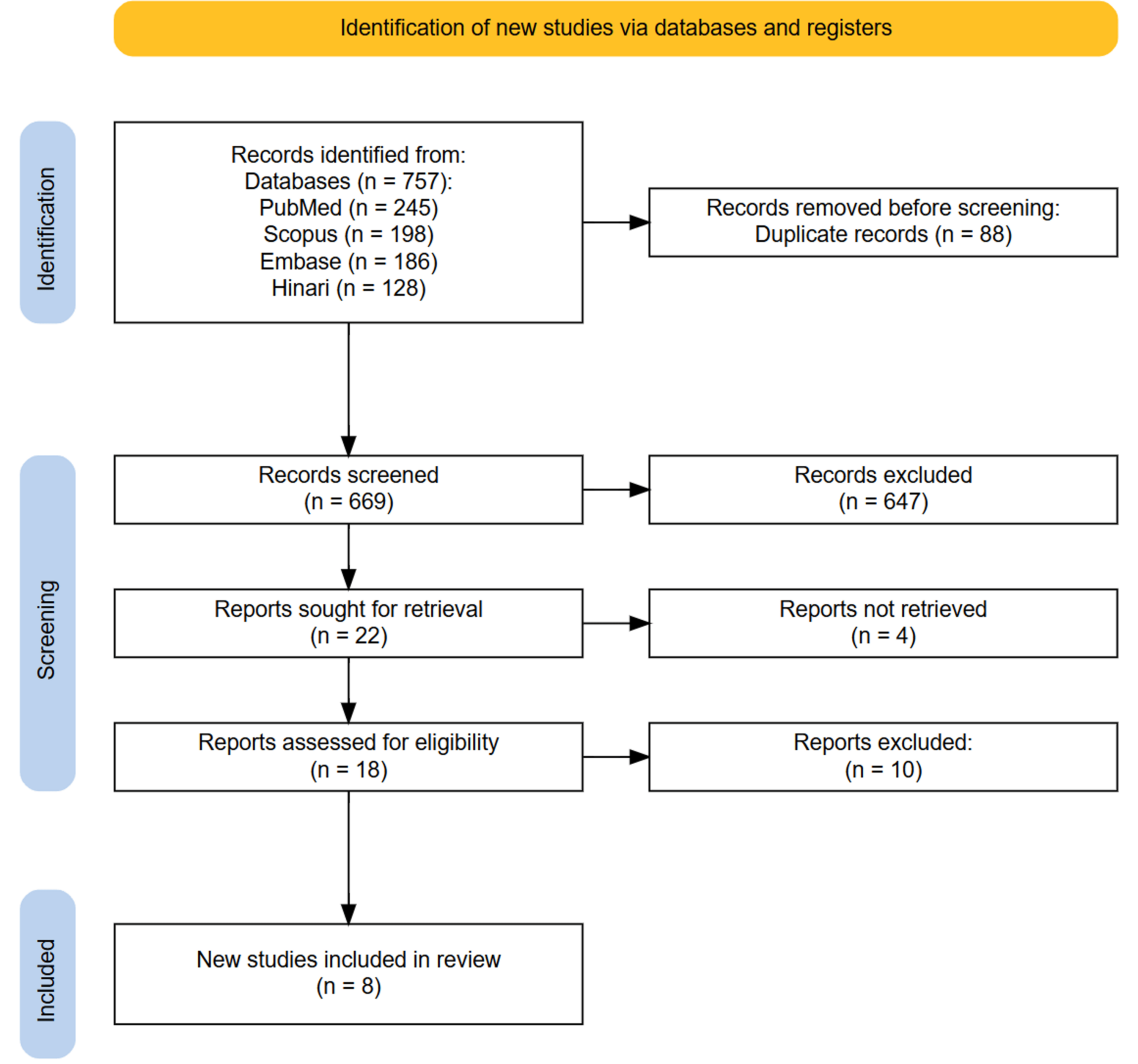
PRISMA diagram illustrating the study selection process. PRISMA, Preferred Reporting Items for Systematic Reviews and Meta-Analyses

A total of 757 records were identified through the initial searches of PubMed, Scopus, Embase, and Hinari databases. After removing 88 duplicate entries, 669 unique records remained for title and abstract screening. During this phase, 647 studies unrelated to IPF, AI, ML, or CT imaging were excluded. As a result, 18 full-text articles were assessed for eligibility based on the predefined inclusion and exclusion criteria. Following a full-text review, eight studies met all inclusion criteria and were included in the final systematic review. The study selection process is summarized in the PRISMA flow diagram (Figure 1).

Study Characteristics

The systematic review analyzed eight studies published between 2017 and 2024 that utilized AI/ML approaches for CT imaging analysis in IPF patients. Most studies employed retrospective cohort designs, with sample sizes ranging from 101 to 1,454 participants. Study populations predominantly featured older adults (mean ages 63-69 years), with a strong male predominance (77%-95% male participants) across all studies. The AI/ML methodologies varied significantly, including DL models, SVMs, and ensemble approaches combining handcrafted radiomics with DL. Five studies focused exclusively on prognostic applications, three studies had both diagnostic and prognostic aims, while one study discussed only the diagnostic applications. All studies utilized HRCT imaging, with slice thicknesses generally below 1.5 mm. Key outcome measures included segmentation accuracy, diagnostic performance metrics (AUC, sensitivity, and specificity), correlation with PF tests, and survival prediction. Common limitations included single-center designs, lack of external validation, retrospective approaches, variability in CT acquisition protocols, and relatively small sample sizes for some subgroups. Despite these limitations, the reviewed studies demonstrated promising performance of AI/ML models for IPF diagnosis and prognosis, often comparable or superior to human expert assessment (Table 1).

**Table 1:** Study characteristics of studies included in this systematic review. IPF, Idiopathic Pulmonary Fibrosis; ILA, Interstitial Lung Abnormalities; CT/HRCT, Computed Tomography/High-Resolution CT; AI, Artificial Intelligence; ML, Machine Learning; CNN, Convolutional Neural Network; FVC, Forced Vital Capacity; CTPF, Computed Tomography Pulmonary Fibrosis; Grad-CAM, Gradient-Weighted Class Activation Mapping; DLCO, Diffusing Capacity for Carbon Monoxide; VC, Vital Capacity; FEV1, Forced Expiratory Volume in one second; TLC, Total Lung Capacity; PaO₂/FiO₂, ratio of arterial oxygen partial pressure to fractional inspired oxygen; PFT, Pulmonary Function Tests; AE-IPF, Acute Exacerbation of IPF; AUC, Area Under the Curve; GGO, Ground-Glass Opacity; CPI, Composite Physiologic Index; PVV, Pulmonary Vessel Volume; HR, Hazard Ratio

Author	Year	Study Design	Sample Size	Population Characteristics	AI/ML Models Used	Type of CT Imaging	Purpose (Diagnostic or Prognostic or Both)	Key Findings	Limitations	Conclusions
Thillai et al. [8]	2024	Prospective cohort	446 patients in the PROFILE cohort + 195 patients in the Cambridge validation cohort	Treatment-naive patients with IPF in PROFILE cohort; median follow-up, 39.1 months; 62.1% died over 5 years	Deep learning-based segmentation models using three-dimensional CNN with UNet architecture	CT scans with a slice thickness of 0.5-5 mm	Prognostic	(1) Successful segmentation in 97.8% of scans; (2) Lung volume showed the strongest correlation with FVC (r = 0.82); (3) Lower lung volume, increased vascular volume, and increased fibrosis volume associated with reduced survival; (4) Longitudinally, decreasing lung volume (HR 3.41) and increasing fibrosis volume (HR 2.23) associated with differential survival	CT scans collected as part of routine clinical care rather than protocolized; limited synchronization between CT and PFT timing; scans not spirometry controlled	Automated models can rapidly segment IPF CT scans, providing prognostic near- and long-term information, which could be used in routine clinical practice or as key trial endpoints.
Maetani et al. [9]	2024	Retrospective cohort	1,454 total (106 IPF, 53 ILA, 1,295 controls)	Male patients with IPF aged ≥40 years, male health checkup subjects were divided into ILA and controls; the IPF group had a higher proportion of former smokers	AIQCT (Fujifilm Corporation, Tokyo, Japan); U-Net architecture for automated segmentation and quantification of lung parenchyma and airway abnormalities	HRCT (0.5 mm/1.25 mm slice thickness), reconstructed with a sharp kernel	Diagnostic and prognostic comparison of central airway structure among IPF, ILA, and controls; assessed the prognostic impact of airway parameters in IPF	(1) Lumen and wall area of intrapulmonary airways were larger in IPF and ILA than in controls; (2) Tracheal tortuosity and curvature were higher in IPF but not ILA; (3) Increased airway wall area is significantly associated with greater mortality in IPF, independent of fibrotic volume	(1) Only male subjects were included; (2) Japanese population only, limiting generalizability; (3) Multiple CT scanners used; (4) Limited smoking data in the health checkup cohort	Central airway lumen and wall areas were enlarged in IPF and ILA, with a weak association with fibrosis. Wall thickening develops early (ILA) and serves as an independent prognostic factor in IPF.
Huang et al. [10]	2024	Retrospective cohort	306 patients (131 internal dataset A, 175 external dataset B)	Mean age: 69.2 years; 77% male; 146 stable IPF, 97 AE-IPF, 63 healthy controls; 54.2% never-smokers; Mean PaO₂/FiO₂: 282.4 ± 129.3	Novel 3D-based deep learning model “SlowFast”; DeepLabV3+ for preprocessing	HRCT chest (slice thickness <1.5 mm)	Diagnostic and prognostic (focus on AE-IPF classification and correlation with PaO₂/FiO₂)	SlowFast model achieved AUC 0.96 and 0.92 on test sets, outperforming radiologists; κw = 0.90 with majority opinion; predicted disease severity via PaO₂/FiO₂	Small sample size; tested only on IPF/AE-IPF/healthy controls; benchmarked against three radiologists	Deep learning model comparable to human readers in classifying AE-IPF; potential for consistent, low-cost patient stratification and decision support.
Wu et al. [11]	2022	Retrospective cohort	206 IPF patients (plus 102 initial cases for model training)	Mean age: 64.1 ± 7.9 years; 95.1% male (196/206); 75.7% with smoking history (156/206)	Deep learning AI model with neural network (lung segmentation network, LSN) with attention mechanism and Squeeze-Excitation Network enhancements	HRCT with 1-2 mm thickness sections, 2 cm section spacing	Prognostic	(1) An AI honeycomb segmentation system was developed to calculate the CT fibrosis score (CTS) as a percentage of honeycomb tissue in the lung; (2) Created a comprehensive CTPF model combining CTS with clinical parameters; (3) The model showed excellent predictive value - AUC index and Briers scores at 1, 2, and 3 years: 74.3.3 (63.2, 85.4), 8.66 (2.4, 14.8); 78 (70.2, 85.9), 16.0.0 (10.1, 22.0); and 72.8.8 (58.3, 87.3), 18.2.2 (11.9, 24.6); (4) CT score is highly correlated with manual radiologist assessment (rs = 0.80, p < 0.01); (5) CT score correlated with lung function parameters	The major limitation of this study is the lack of an external validation cohort to further evaluate the CTPF model	This CTPF model with AI technology can predict mortality risk in IPF precisely.
Sun et al. [12]	2022	Retrospective cohort	101	101 IPF patients (93 males, 8 females); median age 63 years (IQR 58-68); predominantly male (92.1%)	Deep learning-based method for pulmonary segmentation; automatic integration segmentation method for pulmonary vessels	HRCT	Both diagnostic and prognostic (quantified CT features and analyzed correlation with pulmonary function tests)	(1) Total lesion extent showed the strongest negative correlation with DLCO (r = -0.67); (2) Among lesion types, honeycombing showed the strongest correlation with DLCO (r = -0.45); (3) The reticular pattern had the strongest relationship with VC, FVC, FEV1, and TLC; (4) Pulmonary vascular parameters positively correlated with PFTs; (5) Lesion extent negatively correlated with vascular parameters	(1) Limited number of patients due to strict inclusion criteria; (2) Potential confusion between honeycombing and reticular patterns with pulmonary vessels in severe fibrosis cases; (3) Cohort excluded patients with severe disease unable to perform PFTs, limiting generalizability	Quantitative analysis of HRCT features showed a decline in function and vascular destruction with increasing lesion extent. A positive correlation between vascular parameters and pulmonary function was confirmed, indicating the potential of vascular parameters as new objective markers for evaluating IPF severity.
Refaee et al. [13]	2022	Retrospective cohort	474 HRCT scans total (365 for training with 5-fold cross-validation; 109 for external validation)	Mean age 64.10 years ± 9.57 (SD); Included 335 non-IPF ILD patients and 139 IPF patients	Handcrafted radiomics (HCR) using Random Forest classifier, Deep Learning (DL) using 3D Densenet-121, and an ensemble model combining HCR and DL	HRCT with slice thickness <1.5 mm	Diagnostic (to differentiate between IPF and non-IPF interstitial lung diseases)	In 5-fold cross-validation, HCR model accuracy is 76.2 ± 6.8%, DL model 77.9 ± 4.6%, and ensemble model 85.2 ± 2.7%; On the external test set: HCR accuracy 76.1%, DL 77.9%, ensemble 85.3%; The ensemble model outperformed clinicians who achieved a mean accuracy of 66.3 ± 6.7% (p < 0.05); The ensemble model achieved an AUC of 0.917, significantly higher than the HCR model (0.817, p = 0.02) and the DL model (0.823, p = 0.005)	Different CT acquisition and reconstruction settings across datasets may influence HCR feature values, limitations in the interpretability of Grad-CAM results, need for prospective trials in real-world environments, quality of lung segmentation can affect model performance	Deep learning and handcrafted radiomics models can complement each other and serve as useful clinical aids for the diagnosis of IPF and non-IPF ILDs. An ensemble of these models performs better than either model alone and better than clinicians in diagnosis.
Bak et al. [14]	2019	Retrospective cohort	205 patients with IPF	Mean age 65.8 years, 78% male, 31.2% had a surgical lung biopsy, mean FVC 79.0%, mean DLCO 62.9%	Texture-based automated system with Support Vector Machines (SVMs)	Volumetric thin-section CT	Prognostic (identify phenotypes with quantitative CT fibrosis and emphysema features using cluster analysis to assess prognostic impact)	(1) Three distinct radiologic phenotypic clusters were identified using quantitative CT features; (2) Cluster 1 (low fibrosis, low emphysema, high FVC) had a better prognosis than clusters 2 and 3 with higher fibrotic scores; (3) Formula developed (1.5670 - fibrotic score(%) × 0.04737 - emphysema index × 0.00304) to assess the impact of fibrosis and emphysema on lung function; (4) FVC may be preserved in patients with IPF and emphysema despite extensive disease on CT	(1) CT scans not obtained using the same scanner/protocol; (2) Small sample sizes in clusters 2 and 3; (3) Complications like pulmonary hypertension not accounted for; (4) Single-center study requiring validation by prospective studies in larger cohorts	Quantitative CT features of fibrosis and emphysema can classify IPF into discrete subgroups with different prognoses. Prognosis worsens with increased CT fibrosis, and GGO extent may affect outcomes in severe fibrosis. Emphysema can affect FVC measurements. The developed formula helps predict lung function based on the extent of emphysema and fibrosis on CT.
Jacob et al. [15]	2017	Retrospective cohort	283 consecutive patients with IPF	Median age: 67 years; Male/Female: 219/64; Smoking history: 34% never-smokers, 63% ex-smokers, 3% current smokers; Mean follow-up: 30.0 ± 21.5 months; Mortality: 210 (74%) patients died during the study period	CALIPER (Computer-Aided Lung Informatics for Pathology Evaluation and Rating), a quantitative CT algorithm	Non-contrast, supine, volumetric CT scans	Prognostic; mortality prediction using CT and PFT measures	(1) CALIPER-derived pulmonary vessel volume (PVV) was the strongest mortality predictor; (2) Independent mortality predictors: CPI (HR 1.05), PVV (HR 1.23), and CALIPER honeycombing (HR 1.18); (3) The three-group staging model was strongly predictive of mortality; (4) CALIPER parameters outperformed traditional visual CT scores; (5) Quantitative CT variables were better predictors of mortality than visually assessed CT parameters	Lack of external validation cohort	CALIPER-derived parameters, especially PVV, are superior to traditional CT scores for prognosis in IPF. Quantitative tools like CALIPER may enhance staging systems.

Quality Assessment

The methodological quality of the included studies was assessed using the Newcastle-Ottawa Scale (NOS) for observational studies, evaluating three domains: selection (four items), comparability (one item), and outcome (three items). Studies could receive a maximum of nine stars. Out of eight studies, four were of high quality, and four were of moderate quality (Table 2). Common strengths included representative study populations and adequate follow-up periods. However, some studies lost points due to a lack of external validation cohorts, single-center designs, and insufficient control for confounding factors. The retrospective nature of most studies, and male-predominant populations, further limited generalizability. Overall, the evidence base showed moderate quality, with significant methodological heterogeneity across studies.

**Table 2:** Quality assessment of included studies using the Newcastle-Ottawa Scale (NOS) for observational studies. Studies were evaluated across three domains: Selection (maximum 4 stars), Comparability (maximum 2 stars), and Outcome (maximum 3 stars). Total scores ranged from 0-9 stars, with high quality (7-9 stars), moderate quality (4-6 stars), and low quality (0-3 stars). ★ indicates criterion met; ☆ indicates criterion not met or unclear.

Author	Selection (4 Stars)	Comparability (2 Stars)	Outcome (3 Stars)	Total Score	Quality Rating
Thillai et al. [8]	★★★☆	★★	★★★	8/9	High
Maetani et al. [9]	★★☆☆	★☆	★★★	6/9	Moderate
Haung et al. [10]	★★★☆	★★	★★☆	7/9	High
Wu et al. [11]	★★☆☆	★☆	★★☆	5/9	Moderate
Sun et al. [12]	★★☆☆	★☆	★★☆	5/9	Moderate
Refaee et al. [13]	★★★★	★★	★★☆	8/9	High
Bak et al. [14]	★★☆☆	★☆	★★★	6/9	Moderate
Jacob et al. [15]	★★★☆	★★	★★★	8/9	High

Discussion

This systematic review synthesizes the current evidence on AI and ML applications for the diagnosis and prognosis of IPF using CT imaging, highlighting both the promising advances and persistent challenges in this rapidly evolving field. The included studies demonstrate the potential of AI-based approaches to transform several aspects of IPF management, from initial diagnosis to prognostication and treatment response monitoring. A key finding across multiple studies is the superior performance of AI algorithms in quantifying disease extent compared to traditional visual assessment. The work by Wu et al. (2022) and Jacob et al. (2017) showed that AI-derived parameters - particularly measures of fibrotic burden and pulmonary vascular volume - outperformed conventional visual CT scores for prognostication [11,15]. The incorporation of Squeeze-Excitation Network (SENet) enhancements in AI models for IPF detection can potentially improve performance by adaptively recalibrating channel-wise feature responses, allowing the model to capture subtle features and patterns in lung images. SENet can be relatively easily implemented using popular DL libraries such as TensorFlow or PyTorch. In fact, PyTorch has a built-in implementation of SENet, making it straightforward to integrate into a model architecture. This shows that the new models (i.e., computed tomography pulmonary fibrosis (CTPF) model), which combine AI-derived CT pulmonary fibrosis staging with clinical parameters (PF grading), outperformed other models (PF, CT, and GAP (gender, age, physiology) staging) in predicting mortality risk for IPF patients. The AI-derived models' improved performance in predicting mortality risk suggests that the integration of AI-derived quantitative CT measures with clinical parameters can enhance prognostic accuracy - potentially by providing a more comprehensive and objective assessment of disease severity than traditional staging systems. For diagnostic applications, the ensemble model developed by Refaee et al. (2022) achieved significantly higher accuracy than either radiomics or DL approaches alone, and notably outperformed clinicians in differentiating IPF from other ILDs [13]. This highlights the complementary nature of different AI methodologies and suggests that integrated approaches may provide the optimal diagnostic performance. The high diagnostic accuracy (AUC 0.96) of the SlowFast model proposed by Huang et al. (2024) for acute exacerbation detection further underscores the potential clinical utility of AI in critical diagnostic contexts [10].

The consistently strong correlations between AI-quantified CT features and PF tests across multiple studies - Thillai et al. (2024), Wu et al. (2022), Sun et al. (2022) - suggest that AI-derived imaging biomarkers may serve as reliable surrogates for physiological parameters [8,11,12]. This could potentially reduce the need for frequent invasive testing and provide more comprehensive disease assessment, especially in patients unable to perform reliable PF tests due to disease severity or comorbidities. Several studies have identified novel prognostic markers through AI analysis. Maetani et al. (2024) demonstrated that central airway parameters, particularly wall thickening, serve as independent prognostic factors in IPF, while Bak et al. (2019) identified distinct radiologic phenotypes with differential outcomes using texture-based analysis [9,14]. These findings expand our understanding of IPF pathophysiology beyond the traditional focus on parenchymal changes and suggest new avenues for disease monitoring and therapeutic targeting.

The capability of AI algorithms to segment and quantify specific anatomical structures and pathological patterns, demonstrated by Thillai et al. (2024) and Sun et al. (2022), offers opportunities for standardized, objective assessment of disease progression [8,12]. This could be particularly valuable for clinical trials, where reliable endpoints are crucial for evaluating the efficacy of novel therapies. The high correlation (rs = 0.80) between the automated CT fibrosis score and radiologist assessment, reported by Wu et al. (2022), supports the validity of AI-derived metrics as potential trial endpoints [11]. Auto-segmentation uses algorithms to automatically identify and delineate specific structures, organs, tumors, lesions, or regions of interest (ROI) in medical images. By this automated recognition of ROI, radiologists’ time could be significantly freed up, and accurate measurement of disease progression over time could be enabled if suitably auto-segmented images from a particular case are followed up longitudinally. Longitudinal analyses, though limited in number among the included studies, suggest that AI-quantified changes in lung volume and fibrosis extent over time may provide stronger prognostic information than single time-point assessments. Thillai et al. (2024) found that decreasing lung volume (HR 3.41) and increasing fibrosis volume (HR 2.23) were significantly associated with differential survival outcomes, highlighting the potential value of AI for dynamic disease monitoring [8].

The integration of clinical parameters with imaging biomarkers, as demonstrated in the CTPF model by Wu et al. (2022), resulted in enhanced prognostic performance compared to either modality alone [11]. This multimodal approach aligns with the growing recognition that comprehensive IPF assessment requires the integration of imaging, physiological, and clinical data, and suggests that AI may facilitate such integrated analyses in routine clinical practice.

Limitations and future directions

Despite the promising results, several important limitations persist across the current literature. Most studies employed retrospective designs with inherent selection biases, and there was significant heterogeneity in AI methodologies, CT acquisition protocols, and outcome measures, complicating direct comparisons between studies. Sample sizes were generally modest, particularly for subgroup analyses, and predominantly consisted of male patients (>75% in most studies), limiting generalizability to female patients who may exhibit different disease patterns and progression rates. External validation was notably absent in several studies, including Wu et al. (2022), raising concerns about the generalizability of AI models across different patient populations and imaging platforms [11]. The lack of standardized reporting guidelines for AI studies in thoracic imaging further complicates quality assessment and replication of findings. The "black box" nature of many DL algorithms remains a significant barrier to clinical implementation, as clinicians may be reluctant to base decisions on predictions they cannot interpret. Only a few studies, such as Refaee et al. (2022), incorporated explainability techniques like gradient-weighted class activation mapping (Grad-CAM), highlighting an important area for methodological advancement [13].

Future research should prioritize large, prospective, multi-center studies with diverse patient populations and standardized imaging protocols to establish the generalizability of AI approaches. Developing internationally accepted standards for model development, validation, and reporting would facilitate comparison across studies and accelerate clinical translation. Integration of XAI techniques should be emphasized to enhance interpretability and clinical trust. Longitudinal studies with extended follow-up periods are needed to evaluate the predictive value of AI-derived biomarkers for long-term outcomes and treatment response. Additionally, research exploring the integration of AI tools into clinical workflows and their impact on decision-making and patient outcomes would provide crucial evidence for regulatory approval and reimbursement.

As the field advances, studies examining the cost-effectiveness of AI implementation, including potential reductions in healthcare utilization through earlier diagnosis and more precise prognostication, will be essential for broader adoption. Finally, ethical considerations, including algorithm bias, data privacy, and equitable access, must be systematically addressed to ensure that AI technologies benefit all patients with IPF, regardless of demographic or socioeconomic factors.

## Conclusions

This systematic review demonstrates that AI and ML approaches offer promising solutions for enhancing IPF diagnosis and prognosis using CT imaging. AI-derived parameters consistently outperform traditional visual assessment, providing objective quantification of disease extent and novel prognostic biomarkers. The strong correlation between AI-quantified imaging features and pulmonary function supports their potential as reliable surrogate markers for disease progression. Integration of clinical parameters with imaging biomarkers further enhances prognostic performance. However, significant challenges remain, including limited external validation, heterogeneous methodologies, and the "black box" nature of many algorithms. Future research should focus on prospective multi-center studies with standardized protocols, diverse populations, and XAI techniques to facilitate clinical translation. Addressing these challenges could transform IPF management by enabling earlier diagnosis, more precise prognostication, and personalized treatment approaches, ultimately improving outcomes for patients with this devastating disease.
